# A New *β*-Galactosidase from *Pseudomonas tritici* SWRI145 for Efficient Bioproduction of Galactooligosaccharides

**DOI:** 10.3390/foods14173125

**Published:** 2025-09-06

**Authors:** Xiangpeng Jin, Zhuo Cheng, Yulei Zhang, Penka Petrova, Kaloyan Petrov, Wenli Zhang, Wanmeng Mu

**Affiliations:** 1 State Key Laboratory of Food Science and Resources, School of Food Science and Technology, Jiangnan University, Wuxi 214122, China; 2 Institute of Microbiology, Bulgarian Academy of Sciences, 1113 Sofia, Bulgaria; ppetrova@microbio.bas.bg; 3 Institute of Chemical Engineering, Bulgarian Academy of Sciences, 1113 Sofia, Bulgaria; kaloian04@yahoo.com

**Keywords:** galactooligosaccharides, *β*-galactosidase, hydrolysis, transglycosylation

## Abstract

Galactooligosaccharides (GOS) are a class of prebiotic carbohydrates composed of 2 to 8 galactose units linked together and often terminated with a glucose molecule. GOS have attracted significant attention for their health-promoting properties, including the regulation of gut microbiota, promotion of infant health, immune modulation, laxative effects, and potential metabolic benefits. Widely utilized in functional foods, infant formulas, dairy products, and dietary supplements, GOS occur naturally in human milk and are primarily industrially produced through the enzymatic conversion of lactose. *β-*Galactosidase is a crucial enzyme in GOS bioproduction, which exhibits dual functions of hydrolysis and transglycosylation. In this investigation, a novel *β-*galactosidase from *Pseudomonas tritici* SWRI145 (Pstr *β*-galactosidase) was characterized. Biochemical characterization revealed that the enzyme exhibits the highest activity at 50 °C and pH 7.5, with a specific activity of 331.9 U/mg against ONPG. Under optimal reaction conditions (40 °C, pH 8.0, 300 g/L lactose, 0.4 mg/mL enzyme), 134.3 g/L GOS were produced, corresponding to 44.8% GOS yield and 80% substrate conversion. LC-MS analysis confirmed that the main products were GOS with degrees of polymerization (DP) ranging from 2 to 4. To our knowledge, this is the first report of a *Pseudomonas*-derived *β*-galactosidase with demonstrated GOS synthesis capability, highlighting its potential for industrial application.

## 1. Introduction

In recent years, there has been a notable shift in food research towards nutrition and health. Galactooligosaccharides (GOS), which are considered as non-digestible prebiotics when their degree of polymerization (DP) ≥ 3, have garnered considerable attention due to their substantial health benefits. GOS are a type of prebiotic carbohydrates consisting of linked galactose units, usually terminating with a glucose molecule. GOS typically contain 2 to 8 galactose units connected by *β* (1 → 4), *β* (1 → 6), or *β* (1 → 3) glycosidic bonds [1]. GOS exhibit excellent physicochemical properties, including low caloric values of around 1.73 kcal/g, mild clean taste equivalent to 30–60% of sucrose sweetness, and good stability and solubility at high temperatures.

Furthermore, GOS also exhibits numerous special physiological functions, especially in promoting gut health and modulating the immune system [1,2,3,4]. The molecular structure of GOS, particularly the degree of polymerization, and monosaccharide linkage bonds could greatly influence their prebiotic activities [5]. Recent studies have found that GOS and *Lactobacillus reuteri* collaborate synergistically to promote the pentadecanoic acid biosynthesis by *Bacteroides acidifaciens*, thereby reducing gut inflammation and improving barrier dysfunction [6].

GOS are scientifically well-tolerated by the majority of individuals and have been approved for use as food ingredients by the European Food Safety Authority (EFSA) and the Food and Drug Administration (FDA). EFSA has approved the use of GOS in dairy confectionary, cheese, processed cheese, butter, and spreads [7]. In GRAS Notifications 236, GRN 236, FDA gave a “no questions” letter to the first GOS product in term infant formula and selected conventional foods [8]. GOS show extensive application in the food industry, specifically in dairy products, infant formula, dietary supplements, and pet food. In infant formulas, GOS play a crucial role as a functional ingredient, stimulating the growth of probiotics like *Bifidobacterium* spp. in the infant’s intestines and enhancing the infant’s developing immune system [9]. In vanilla ice creams, 3.0% (*wt*/*wt*) GOS addition could enhance the firmness, lower the freezing point, and decrease melting rates, resulting in improved texture and creaminess [10]. Replacing sucrose with GOS in chocolate can not only reduce the postprandial glycemic index but also improve sensory qualities through increased viscosity, hardness, and yield strength [11]. The properties of whey protein powder are significantly improved by wet heating at 90 °C in combination with GOS, resulting in improved solubility, emulsification, foam stability, and antioxidant activity [12]. Supplementing GOS into yogurt can not only enhance its textural properties, such as firmness, creaminess, and smoothness [13], but also promote the absorption and retention of calcium, thereby enhancing bone development during the growth stage [14]. Furthermore, many studies have confirmed that the addition of GOS in pet feeds can help regulate intestinal health, improve bowel movements, stimulate animal growth, and minimize hair loss [15,16,17].

Naturally present in human milk, the commercial industrial production of GOS usually includes the use of *β-*galactosidase enzymes (*β-*galactosidase, EC 3.2.1.23), which convert lactose into a mix of GOS along with residual lactose, galactose, and glucose. *β-*Galactosidase is a bifunctional enzyme with hydrolytic and transglycosidic activities, and it has emerged as one of the most widely employed enzymes in the food industry [18]. *β-*Galactosidase is widely present in nature and can be derived from various microorganisms, including yeast, bacteria, and filamentous fungi [19]. Usually, the enzyme sources of *β-*galactosidase play crucial roles in the production of GOS, influencing factors such as yields, polymerization degrees, and bond types. When utilizing the *β-*galactosidase from *Aspergillus oryzae*, *Kluyveromyces lactis*, and *Bacillus circulans* as biocatalysts, the maximum GOS yields can reach 19.5%, 34.9%, and 48.3%, respectively [20]. The *β-*galactosidase from *Aspergillus oryzae* primarily yields GOS with *β* (1 → 6) bonds, while the *β-*galactosidases from *Bacillus circulans* and *Kluyveromyces lactis* mainly generate GOS with *β* (1 → 4) bonds [21]. The *β-*galactosidase from *Lactobacillus bulgaricus* can specifically produce GOS with DP of 3 specifically and efficiently, achieving a yield ratio of 34% [22]. Moreover, reaction conditions such as temperature, pH, substrate concentration (lactose), and the amount of *β-*galactosidase enzyme significantly affect the GOS yield.

The present work aims to identify new natural *β*-galactosidases produced by bacteria that have not been utilized as GOS producers to date. A candidate enzyme from *Pseudomonas tritici* was identified through in silico screening using the EnzymeMiner platform and subsequently characterized. *Pseudomonas tritici* SWRI145, a species belonging to the *P. fluorescens* subgroup, was isolated from the rhizosphere of wheat (cultivar Zarin), Salmas, Iran, and has a G + C content of 59.99 mol% [23]. In addition, the effects of several key reaction parameters on GOS synthesis were systematically investigated, including pH, temperature, enzyme concentration, and lactose concentration. Given the lack of data on transgalactosylation-active enzymes within the *Pseudomonas* genus, this work provides initial evidence that members of this group may also serve as viable sources for GOS biosynthesis. These findings demonstrated that Pstr *β-*galactosidase possessed a high GOS conversion efficiency, indicating its promising application as a biocatalyst in industrial-scale GOS production.

## 2. Materials and Methods

### 2.1. Plasmid, Strains, and Chemicals

O-Nitrophenyl-β-D-galactopyranoside (ONPG) was obtained from Shanghai Yuanye Bio-Technology Co., Ltd. (Shanghai, China); standard galactooligosaccharides (GOS) were sourced from Shanghai Macklin Biochemical Technology Co., Ltd. (Shanghai, China). The substrate lactose (α-Lactose monohydrate) was supplied by Sinopharm Chemical Reagent Co., Ltd. (Shanghai, China). The Ni^2+^ affinity resin column used for protein purification was purchased from Cytiva (Uppsala, Sweden). The Bradford protein assay kit was acquired from Beyotime Biotechnology Co., Ltd. (Shanghai, China). Additional chemicals and reagents were provided by Sigma-Aldrich (St. Louis, MO, USA) and Sinopharm (Shanghai, China). Plasmid construction and sequencing were carried out by Sangon Biotech Co., Ltd. (Shanghai, China).

### 2.2. Molecular Cloning of Pstr β-Gal

The gene encoding *β-*galactosidase from *Pseudomonas tritici* SWRI145 (Pstr *β-*galactosidase), previously deposited in the GenBank database (accession number MBC3295486.1), was synthesized, amplified, and subcloned into the pET-28a(+) expression vector using *Nco*I and *Xho*I restriction sites. To facilitate purification, a C-terminal 6 × His-tag was introduced to the gene construct. The recombinant plasmid was first replicated in *E. coli* JM109 for cloning purposes and subsequently transferred into *E. coli* BL21(DE3) for the expression of the target protein.

### 2.3. Three-Dimensional Modeling of Pstr β-Galactosidase

The three-dimensional structure of Pstr *β-*galactosidase was predicted using AlphaFold3 for homology modeling. The structural quality of the predicted model was assessed using the SAVES server suite (v6.1, The UCLA-DOE Institute for Genomics and Proteomics, Los Angeles, CA, USA).

The alignment of the Pstr *β*-galactosidase structure modelled by α-Fold3 with the crystal structure of *β*-galactosidases derived from *Escherichia coli.*

### 2.4. Protein Overexpression and Purification of Pstr β-Galactosidase

Recombinant *E. coli* cells containing the plasmid encoding the Pstr *β-*galactosidase gene were cultured in a 500 mL shake flask with 200 mL LB medium (yeast extract 5 g/L, NaCl 10 g/L, tryptone 10 g/L), supplemented with 100 μg/mL kanamycin. Cultures were incubated at 37 °C with agitation at 200 rpm. When the OD_600_ reached 0.6–0.8, isopropyl *β*-D-1-thiogalactopyranoside (IPTG) was added to a final concentration of 1 mM to induce Pstr *β*-gal expression. The induction was carried out at 28 °C for 8 h. Following induction, cells were collected by centrifugation at 8000× *g* for 5 min at 4 °C.

The harvested cells were lysed by sonication, and the supernatant was clarified via filtration and centrifugation. The recombinant protein, containing a C-terminal 6 × His tag, was purified using Ni^2+^-nitrilotriacetic acid (Ni-NTA) affinity chromatography with a column size of 1.6 cm × 20 cm. The purification process employed three buffer systems: a binding buffer composed of 50 mM HEPES and 500 mM NaCl (pH 7.5), a washing buffer supplemented with an additional 50 mM imidazole, and an elution buffer containing an additional 500 mM imidazole. His-tagged proteins bind selectively while untagged impurities flow through in the environment of the binding buffer, and washing buffer was used to remove weakly bound contaminants. Finally, the target protein is eluted and collected by applying an elution buffer containing 500 mM imidazole. Following purification, to eliminate imidazole and metal ions, the eluted protein was dialyzed sequentially against dialysis buffers, including a solution composed of 50 mM HEPES buffer with or without 10 mM EDTA, respectively. The purity of the recombinant Pstr *β-*galactosidase was assessed by SDS-PAGE using an 8% Bis-Tris SDS-PAGE Gel. Protein concentration was quantified using the Bradford protein assay [24].

### 2.5. Hydrolytic Activity Assay of Pstr β-Galactosidase

The enzymatic assay was carried out in a reaction mixture containing 15 mM ONPG 130 and 50 mM HEPES buffer (pH 7.5), supplemented with 10 μg/mL of purified Pstr *β*-gal. The reaction was executed at 50 °C for 5 min, and subsequently terminated by adding 1 mL of 1 M Na_2_CO_3_ to ensure complete enzyme inactivation. The amount of ONP was quantified at OD_420_ using a Tecan Infinite 200 pro microplate reader. One unit (U) of hydrolytic enzyme activity was defined as the amount of enzyme required to catalyze the release of 1 μmol of ONP per minute under the specified conditions.

### 2.6. Effects of Temperature, pH and Metal Ions

To evaluate the effect of temperature on enzyme activity, reactions were conducted in 50 mM HEPES buffer (pH 7.5) for 5 min, with 15 mM ONPG and 10 μg/mL of purified Pstr *β-*galactosidase. The assays were carried out over a temperature range of 40–60 °C. For pH dependence analysis, enzymatic reactions were performed at the optimal temperature of 50 °C, using the following buffer systems (50 mM): HAC-NaAC buffer (pH 5.5–6.0), PBS buffer (pH 6.0–7.0), HEPES buffer (pH 7.0–8.0), and Tris-HCl buffer (pH 8.0–9.0). The influence of metal ions was assessed by supplementing the reaction mixture with 1 mM of various metal ions, including Na^+^, K^+^, Co^2+^, Mn^2+^, Cu^2+^, Mg^2+^, Zn^2+^, Ca^2+^, Ni^2+^, and Fe^3+^. The kinetic parameters of Pstr *β-*galactosidase toward ONPG were evaluated by measuring enzymatic activity at varying substrate concentrations, ranging from 0.3 mM to 60 mM, under standard assay conditions. The Lineweaver–Burk plot has been used to calculate kinetic parameters of Michaelis constant (*K*_m_) and turnover number (*k*_cat_). The *k*_cat_ = *V*_max_/[S] was calculated as the ratio of *V*_max_ to the molar concentration of enzyme [S]. All other assay conditions were maintained as described in the standard activity measurement protocol.

To assess the thermostability of Pstr β-galactosidase, both residual enzymatic activities and melting temperature (T_m_) were evaluated. In the residual activity assay, purified enzyme samples were exposed to incubation in 50 mM HEPES buffer (pH 7.5) at temperatures of 30 °C, 35 °C, and 40 °C. At defined time intervals, samples were withdrawn, and the enzymatic activity was measured under standard assay conditions. The activity of the non-incubated sample was defined as 100% and used as a reference to calculate relative activity. The T_m_ values were determined using a Nano DSC III (TA Instruments, New Castle, DE, USA). The measurement was performed with 1 mg/mL of purified Pstr β-galactosidase, a heating rate of 1 °C/min, a pressure of 3 atm, and a scan temperature range of 20–85 °C.

### 2.7. Optimization of GOS Bioproduction

To optimize the reaction conditions for maximizing GOS yield, key parameters including temperature, pH, substrate concentration, and enzyme dosage were systematically investigated. To evaluate the influence of temperature, enzymatic reactions were performed in a 1 L reaction mixture containing 50 mM HEPES buffer (pH 8.0), 300 g/L lactose, 1 mM Mg^2+^, and 0.4 mg/mL purified Pstr *β-*galactosidase, within a temperature range of 35–45 °C. The effect of pH was assessed under the same conditions by varying the buffer pH from 7.0 to 8.5. To examine the impact of substrate concentration, lactose was applied at 100, 200, and 300 g/L. To evaluate the role of enzyme dosage, reactions were carried out with Pstr *β-*galactosidase concentrations ranging from 0.05 to 0.5 mg/mL. The quantity of GOS is determined by subtracting the amount of monosaccharides (glucose and galactose) and lactose from the total sugar content [25]. The lactose amount was quantified utilizing HPLC equipped with a refractive index detector. Separation was performed on an XBridge BEH Amide column (4.6 × 250 mm). The mobile phase consisted of 80% acetonitrile, 20% ultrapure water, and 0.1% ammonia solution, delivered at a flow rate of 1.0 mL/min. The column temperature was set at 35 °C throughout the analysis. For the measurement of glucose and galactose contents, the same HPLC-RID system was employed with a different column. Separation was performed on a Rezex^TM^ ROA-Organic Acid H^+^ (8%) column (300 × 7.8 mm), using a mobile phase of 5 mM sulfuric acid at a flow rate of 0.6 mL/min, with an operating temperature of 60 °C.

### 2.8. Analytical Methods

The product mixture was analyzed by HPLC, following the previously stated procedure, to measure various sugar contents. The products were obtained using 300 g/L lactose as the substrate and 0.4 mg/mL Pstr *β-*galactosidase as the catalyst, supplemented with 1 mM Mg^2+^, at 40 °C and pH 8.0 for 8 h. To further analyze the composition of oligosaccharides in the reaction products, a liquid chromatograph mass spectrometer (LC-MS) was employed. For LC-MS analysis, liquid chromatography was performed using a Waters Acquity UPLC system equipped with an ACQUITY UPLC BEH Amide column (1.0 × 150 mm, 1.7 μm). The mobile phase consisted of 80% acetonitrile, 20% ultrapure water, and 0.1% ammonium hydroxide. Chromatographic separation was performed at a flow rate of 0.3 mL/min, with the column maintained at 45 °C. Each run involved injecting a 5 μL sample aliquot. MS analysis was conducted on a Waters ESI-Q-TOF-MS system (Milford, MA, USA) operating in positive electrospray ionization (ESI^+^) mode. The collision energy was set at 6/20 eV, and mass spectra were acquired over 50–1500 *m*/*z*.

All experiments of this study were performed in triplicate, and data are presented as mean ± SD. Statistical significance was assessed by analysis of variance (ANOVA), with a *p*-value < 0.05 considered statistically significant.

## 3. Results and Discussion

### 3.1. Amino Acid Sequence and Structure Analysis

Due to its high GOS production yield of 51.7%, *β-*galactosidase from *Enterobacter cloacae* served as the reference [26], and the key amino acid residues E463, E539, and H542 were used as probes for potential *β-*galactosidase sequence searches through the EnzymeMiner online server [27]. Subsequently, the *β-*galactosidase derived from *Pseudomonas tritici* was screened and selected for GOS production. As shown in Figure S1, the amino acid sequence similarity between Pstr *β-*galactosidase (MBC3295486) and *E. cloacae β-*galactosidase (WFG05994.1) was determined to be 79.1%. According to current taxonomy and genomic databases, *Pseudomonas tritici* SWRI145 is the type strain of its species, recently reported by Girard et al. [23]. Although the complete genome of *Pseudomonas tritici* strain SWRI145 is available, no data have confirmed its ability to produce GOS so far. Moreover, GOS production has not been known for any member of the genus *Pseudomonas* until now. Therefore, a significant finding of this study is the discovery of the first *β-*galactosidase from a *Pseudomonas* species displaying transgalactosidase activity. In this context, the study makes a pioneering contribution by establishing the first functional link between *Pseudomonas β-*galactosidase and the production of GOS, thereby filling a critical knowledge gap and opening new avenues for industrial applications and evolutionary enzymology within the genus *Pseudomonas*.

**Figure 1:**
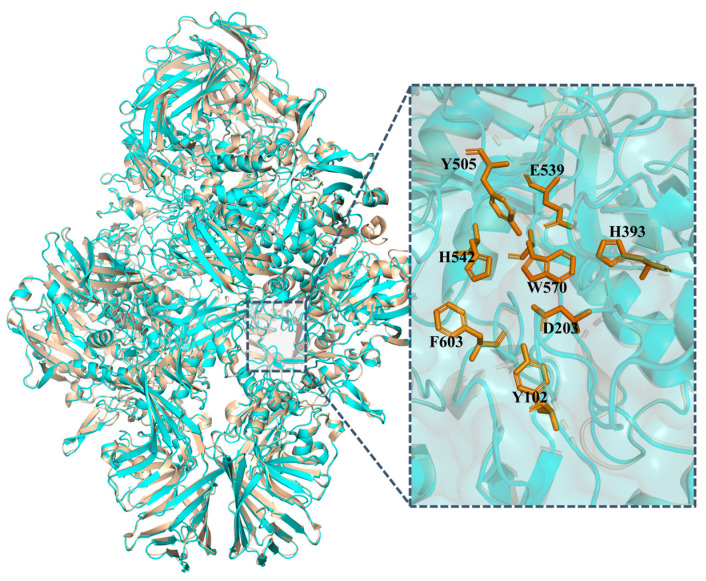
Structural superposition of the predicted Pstr *β-*galactosidase model (cyan) with the crystal structure of *E. coli β-*galactosidase (PDB: 1JYN, yellow).

Evaluation results of AlphaFold3 showed that 83.79% of the residues achieved an average 3D-1D score ≥ 0.1, indicating a high level of model reliability. Additionally, the Ramachandran plot analysis revealed that the vast majority of amino acid residues occupied energetically favorable regions, suggesting that the overall conformational geometry was reasonable (Figure S2). At present, the crystal structures of *β-*galactosidases from several glycoside hydrolase (GH) families have been resolved, including the GH2 *β-*galactosidase from *Escherichia coli* (PDB: 1JYN), the GH35 *β-*galactosidase from *Penicillium* species (PDB: 1TG7), and the GH42 *β-*galactosidase from *Marinomonas* ef1 (PDB: 6Y2K) [28]. Among these, Pstr *β-*galactosidase exhibited the highest sequence and structural similarity to the GH2 family *E. coli β-*galactosidase, with 66.1% amino acid sequence identity. Structural superposition of the AlphaFold3-predicted Pstr *β-*galactosidase model and the *E. coli β-*galactosidase crystal structure revealed a close resemblance (Figure 1), with a root-mean-square deviation (RMSD) of 0.265. Moreover, key catalytic site residues in Pstr *β-*galactosidase, including Y102, D203, H393, Y505, E539, H542, F603, and W570, were found to be highly conserved.

### 3.2. Overexpression and Purification

The gene encoding the putative *β-*galactosidase from *Pseudomonas tritici* SWRI145 (GenBank accession number: ABWQF010000020.1) consisted of 3087 nucleotides and encoded 1028 amino acids. A synthetic version of the gene, which included a C-terminal 6 × His tag, was inserted into the pET-28a(+) expression vector using the *Nco*I and *Xho*I restriction sites (Figure S3). Subsequently, the recombinant plasmid was introduced into *E. coli* BL21(DE3) for the heterologous expression of the target protein, induced by IPTG. The purified recombinant Pstr *β-*galactosidase was obtained through Ni^2+^-affinity chromatography, and its purity was assessed using SDS-PAGE. The theoretical isoelectric point (pI) and molecular weight (Mw) of the protein were predicted to be 5.36 and 116.3 kDa, respectively, based on ExPASy ProtParam analysis [29]. The protein pI is calculated using pK values of different amino acids, while the Mw is calculated by adding the average isotopic masses of amino acids in the protein and the average isotopic mass of one water molecule. This prediction was supported by SDS-PAGE analysis, which showed a clear band at approximately 120 kDa (Figure S3).

### 3.3. Biochemical Characterization of Pstr β-Galactosidase

**Figure 2:**
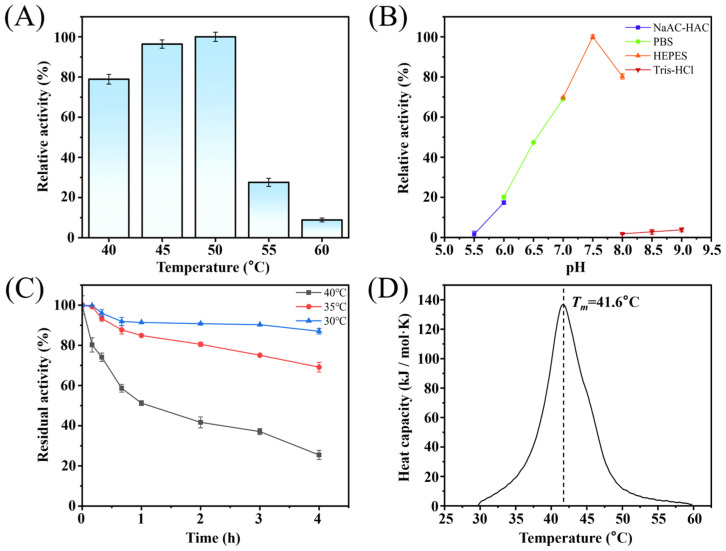
Characterization of Pstr *β-*galactosidase. (**A**) Effect of temperature. (**B**) Effect of pH. (**C**) Thermostability of Pstr *β-*galactosidase. (**D**) Structural stability of Pstr *β-*galactosidase.

To investigate the impact of temperature on Pstr *β-*galactosidase activity, enzymatic assays were conducted over a temperature range of 40–60 °C in HEPES buffer (pH 7.5) containing 1 mM Mg^2+^. The reactions were carried out for 5 min using 15 mM ONPG as the substrate and 10 μg/mL of purified Pstr *β-*galactosidase. As shown in Figure 2A, the enzyme displayed maximum catalytic activity at 50 °C. Notably, more than 78.9% of the maximal activity was retained within the 40–50 °C range. However, enzyme activity declined markedly at temperatures above 50 °C, with relative activity reduced to 27.6% at 55 °C. In industrial GOS production, elevated temperatures were typically favored, as they could facilitate transgalactosylation reactions, enhance lactose solubility, reduce the viscosity of the reaction system, and inhibit microbial growth [1].

To evaluate the influence of pH on Pstr *β-*galactosidase activity, the enzymatic assays were carried out at 50 °C across a pH range of 5.5 to 9.0, using four different buffer systems: HAC-NaAC buffer (pH 5.5–6.0), phosphate-buffered saline (PBS, pH 6.0–7.0), HEPES buffer (pH 7.0–8.0), and Tris-HCl buffer (pH 8.0–9.0). As illustrated in Figure 2B, the enzyme exhibited optimal activity at pH 7.5 in HEPES buffer. Between pH 7.0 and 8.0 (HEPES buffer), more than 69.7% of the maximal activity was maintained. However, outside this optimal pH range, there was a significant decrease in enzymatic activity. Besides the influence of pH, differences in buffer systems also affect the enzyme activity, Pstr *β-*galactosidase exhibits 69.7% of the maximal activity in HEPES buffer, whereas only 1.9% activity is retained in Tris-HCl buffer.

To assess the influence of metal ions on the catalytic activity of Pstr *β-*galactosidase, various metal ions (1 mM final concentration), including Na^+^, K^+^, Co^2+^, Mn^2+^, Cu^2+^, Mg^2+^, Zn^2+^, Ca^2+^, Ni^2+^, and Fe^3+^, were individually added to the reaction system (Table 1).

It was observed that Mg^2+^ significantly enhanced the enzymatic activity by 25.1% compared to the control, which is consistent with findings reported for other *β-*galactosidases [30]. In contrast, all other tested ions exhibited varying degrees of inhibition. Specifically, the presence of K^+^, Fe^3+^, Ca^2+^, Na^+^, and Mn^2+^ reduced the relative activity to 75.6%, 71.2%, 60.7%, 59.4%, and 45.2%, respectively. Notably, Co^2+^, Cu^2+^, Zn^2+^, and Ni^2+^ led to almost complete loss of enzyme activity.

The kinetic properties of *Pstr β-*galactosidase toward ONPG were determined by performing enzymatic assays under standard conditions with substrate concentrations ranging from 0.3 mM to 60 mM. The calculated kinetic parameters included a Michaelis constant (*K*_m_) of 3.0 mM, a turnover number (*k*_cat_) of 38638.8 min^−1^, and a catalytic efficiency (*k*_cat_/*K*_m_) of 12879.6 mM^−1^·min^−1^. Under the optimal reaction conditions (pH 7.5 and 50 °C), Pstr *β-*galactosidase exhibited the highest specific activity of 331.9 U/mg towards ONPG.

To assess the thermostability of Pstr *β-*galactosidase, the purified enzyme was incubated at temperatures ranging from 30 °C to 40 °C. As depicted in Figure 2C, it retained 87.0% and 69.2% of its initial activity after four hours of incubation at 30 °C and 35 °C, respectively.

However, thermostability significantly decreased at higher temperatures. At 40 °C, only 25.5% of the initial activity remained after 4 h, with a calculated half-life (*t*_1/2_) of approximately 1.1 h. In addition, the structural stability of the enzyme was assessed using Nano-DSC, revealing a *T*_m_ value of 41.6 °C (Figure 2D). Many studies have confirmed that metal ions not only affected the catalytic efficiency but also played vital roles in enhancing the thermostability and structural stability of many sugar enzymes, such as ketose 3-isomerase [31] and alginate lyase [32]. However, in the case of Pstr *β-*galactosidase, the presence of metal ions (Mg^2+^) appeared to exert minimal influence on the *t*_1/2_ and *T*_m_ values of Pstr *β-*galactosidase (Figure S4).

### 3.4. Bioconversion of GOS from Lactose

**Figure 3:**
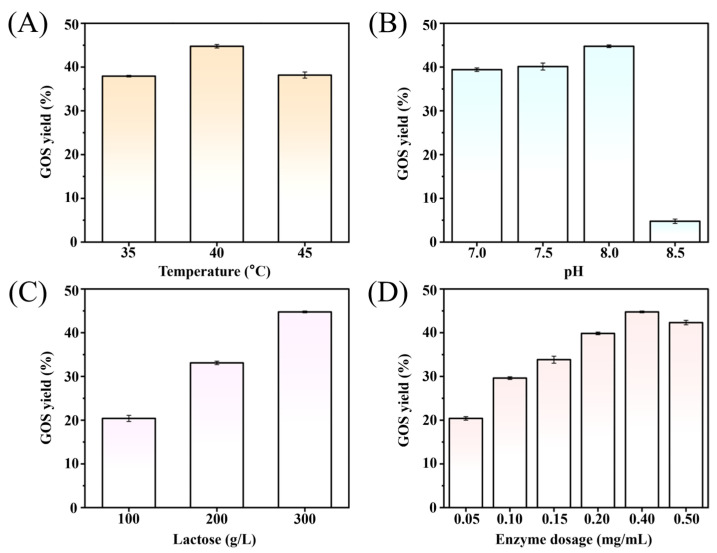
The optimization of GOS production conditions. Temperature of 35–45 °C (**A**), pH of 7.0–8.5 (**B**), substrate concentration of 100–300 g/L (**C**), and enzyme dosage of 0.05–0.5 mg/mL (**D**).

In addition to the *β-*galactosidase sources, the reaction parameters including temperature, pH, substrate concentration, and enzyme dosage, were found to affect the GOS yield significantly [33]. To enhance GOS yield, the reaction conditions mentioned above were optimized. GOS content was determined by subtracting the monosaccharides (glucose and galactose) and residual lactose from the total sugar concentration, and the GOS yield was expressed as the ratio of GOS to total sugars. Optimal reaction conditions of the 1 L reaction mixture were identified as 40 °C, pH 8.0, 300 g/L lactose, and 0.4 g/L enzyme dosage, under which a maximum GOS yield of 44.8% was achieved. As depicted in Figure 3, temperature and pH exhibited only a modest effect on GOS production, with yields ranging from 37.9% to 44.8% (35–45 °C, pH 7.0–8.0). In contrast, substrate concentration and enzyme dosage had a more significant influence on conversion efficiency. An increase in lactose concentration from 100 g/L to 300 g/L and enzyme concentration from 0.05 mg/mL to 0.4 mg/mL resulted in a yield improvement from 20.4% to 44.8%. However, further increases in enzyme dosage beyond 0.4 mg/mL led to a slight decline in GOS yield, dropping to 42.3%.

### 3.5. In Vitro GOS Production

GOS production typically achieved yields ranging from 30% to 40% [34]. Most commercial *β-*galactosidases used in GOS production were derived from *Aspergillus niger* (a filamentous fungus), *Kluyveromyces lactis* (a yeast), *Bifidobacterium bifidum* (a bacterium) and *Bacillus circulans* (a bacterium), with reported GOS yields of 19.5%, 34.9%, 53.8% and 63.8%, respectively [20,35,36]. In recent years, advancements in targeted screening and molecular engineering have led to the discovery of several wild-type and mutant *β-*galactosidases with high GOS yield. For example, the *β-*galactosidase from *E. cloacae* achieved a GOS yield of 51.73% when utilizing 380 g/L lactose as the substrate. Moreover, a mutant variant, H542V, engineered through rational design, exhibited an impressive GOS yield of 67.08% [26]. And the *β-*galactosidase from *Thermotoga maritima* achieved a GOS yield of 72.1% when utilizing 570 g/L lactose as the substrate, showing the highest yield among the reported *β-*galactosidases [37]. Using the glucose re-tunneling strategy, two mutants of *B. circulans* (BglD) *β-*galactosidase, T215Y and T473Y, displayed enhanced GOS production. Compared to the wild-type BglD *β-*galactosidase, which yielded 52.1%, the mutants achieved increased yields of 57.2% and 57.6%, respectively [38]. Through site-directed mutagenesis, two mutants of *Sulfolobus solfataricus β-*galactosidase, F359Q and F441Y, were generated. These mutants exhibited improved GOS yields of 58.3% and 51.7%, respectively, compared to the original yield of 50.9% [39].

**Figure 4:**
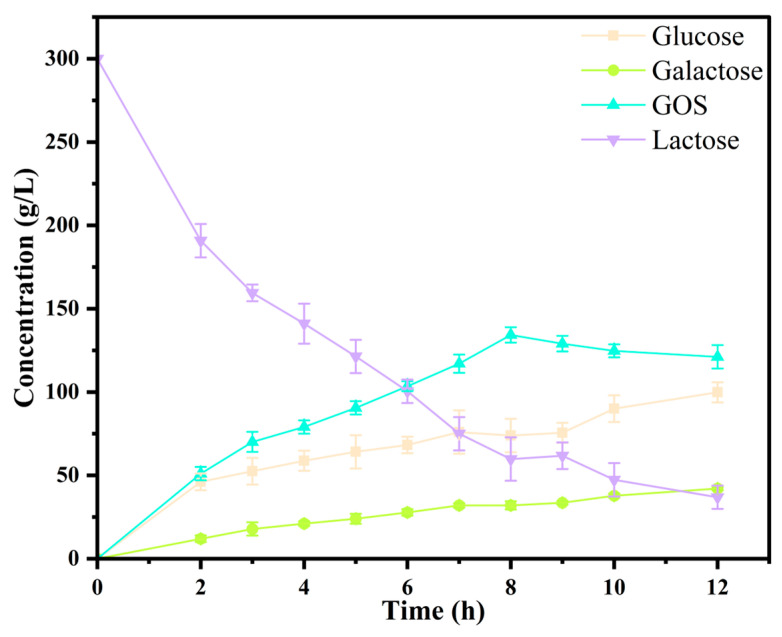
Time courses of enzymatic synthesis of GOS from lactose by Pstr *β-*galactosidase, including the concentration of lactose, GOS, galactose, and glucose.

The time evolution of reaction products was monitored using HPLC analysis. As illustrated in Figure 4, lactose concentration steadily declined over the course of the reaction, accompanied by a continuous increase in the monosaccharide products, glucose and galactose. GOS concentration initially increased, reaching its peak at 8 h with a maximum yield of 44.8%. Following this point, a gradual decline in GOS content was observed. At the time of maximum GOS production, 134.3 g/L of GOS was generated, while the corresponding concentrations of residual lactose, glucose, and galactose were 59.8 g/L, 73.9 g/L, and 32.0 g/L, respectively. The overall lactose conversion rate at this stage was approximately 80.0%.

**Figure 5:**
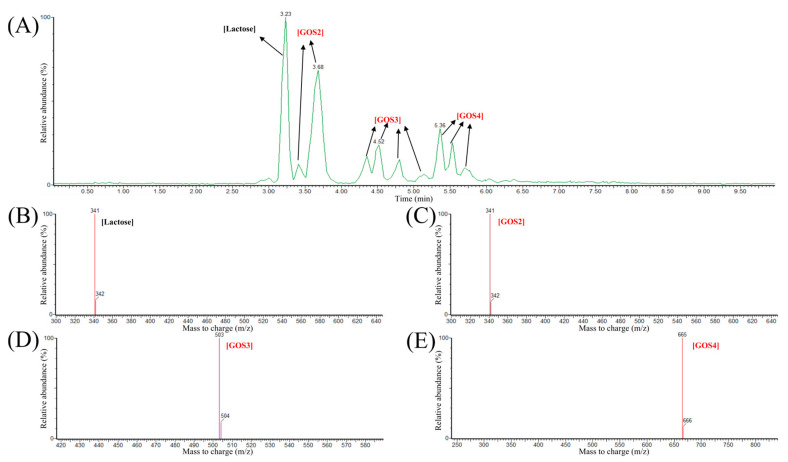
Analysis of Pstr *β*-galactosidase reaction products using LC-MS. (**A**) LC-MS analysis of the products. (**B**–**E**) MS analysis of the products at peaks of 3.23 min (**B**), 3.68 min (**C**), 4.52 min (**D**), and 5.36 min (**E**).

LC-MS further characterized the composition of GOS products. As shown in Figure 5A, LC analysis revealed distinct peaks at retention times of 3.23, 3.68, 4.52, and 5.36 min. The corresponding mass spectra (Figure 5B–E) confirmed the molecular ions of lactose and GOS oligomers. Specifically, the m/z ratios identified were 341.11 for lactose, 341.11 for GOS2, 503.16 for GOS3, and 665.22 for GOS4. Based on LC-MS results, the GOS products produced by Pstr *β-*galactosidase mainly consisted of DP 2 to 4 galactooligosaccharides (GOS2–GOS4). While the Pstr *β-*galactosidase mainly produced GOS2–GOS4, the *β-*galactosidase derived from *Bifidobacterium bifidum* produced a higher percentage of GOS5-GOS6 [35], and GOS7 had been detected in the product of the commercial *β-*galactosidase derived from *Bacillus circulans* [40].

## 4. Conclusions

In this study, a new *β-*galactosidase from *Pseudomonas tritici* SWRI145 was identified via computational screening using the EnzymeMiner server. The *E. cloacae β-*galactosidase was used as the template and its key catalytic residues E463, E539, and H542 were taken as probes. The recombinant Pstr *β-*galactosidase exhibited a specific activity of 331.9 U/mg towards ONPG at pH 7.5 and 50 °C with the addition of 1 mM Mg^2+^. Its stability analysis revealed a *t*_1/2_ of 1.1 h at 40 °C and *T*_m_ value of 41.6 °C, with metal ions exerting minimal influence on its structural stability. Upon optimizing key reaction parameters, including temperature, pH, substrate concentration, and enzyme dosage, 134.3 g/L of GOS could be obtained from an initial lactose concentration of 300 g/L, corresponding to a yield of 44.8% and a lactose conversion efficiency of 80.0%. Further LC-MS analysis confirmed that the main oligosaccharide products were GOS2 to GOS4. Collectively, these findings suggested the promising application potential of Pstr *β-*galactosidase in the enzymatic synthesis of GOS. Furthermore, the Pstr *β-*galactosidase, a new natural *β*-galactosidase produced by *Pseudomonas tritici* SWRI145, has the potential to be served as an efficient, cost-effective, and multifunctional biocatalyst for GOS production in the food industry, particularly in the development of functional foods.

## Figures and Tables

**Table 1:** Effect of metal ions on the activity of recombinant Pstr *β-*galactosidase.

Metal Ion (1 mM)	Relative Activity (%)
None	100 ± 3.6
K^+^	75.6 ± 2.1
Na^+^	59.4 ± 1.3
Mg^2+^	125.1 ± 4.7
Ca^2+^	60.7 ± 1.2
Mn^2+^	45.2 ± 1.7
Co^2+^	3.1 ± 0.3
Ni^2+^	2.4 ± 0.7
Cu^2+^	0.6 ± 0.1
Zn^2+^	1 ± 0.3
Fe^3+^	71.2 ± 2.3

## Data Availability

All datasets generated for this study are included in the article.

## Supplementary Materials

The following supporting information can be downloaded at: https://www.mdpi.com/article/10.3390/foods14173125/s1, Figure S1: Multiple sequence alignment of Pstr *β-*galactosidase with selected representative *β-*galactosidases. Sequence 1 corresponds to *Escherichia coli β-*galactosidase (NCBI accession: WP_000177906.1), Sequence 2 represents Pstr *β-*galactosidase (NCBI accession: MBC3295486.1), and Sequence 3 is derived from *Enterobacter cloacae β-*galactosidase (NCBI accession: WFG05994.1). Red asterisks indicate conserved catalytic residues; Figure S2: The accuracy evaluation of Pstr *β-*galactosidase modelling. (A) Ramachandran plot analysis of Pstr *β-*galactosidase modelling (B) Verify3D analysis of Pstr *β-*galactosidase modelling; Figure S3: The gene map of the recombinant plasmid pET28a-Pstr. (B) SDS-PAGE analysis of Pstr *β-*galactosidase. Lane M, protein markers; lane 1, the purified recombinant Pstr *β-*galactosidase; Figure S4: The influence of the metal ion (Mg^2+^) on the thermostability of Pstr *β*-galactosidase.
