# Supplementary material for: A New β-Galactosidase from Pseudomonas tritici SWRI145 for Efficient Bioproduction of Galactooligosaccharides
Source: Foods. 2025 Sep 6;14(17):3125. doi: 10.3390/foods14173125 (PMC12428694; doi:10.3390/foods14173125)
Supplement: Supplementary file 1 [file foods-14-03125-s001.zip › foods-3789171-supplementary.pdf]

Supplementary Materials

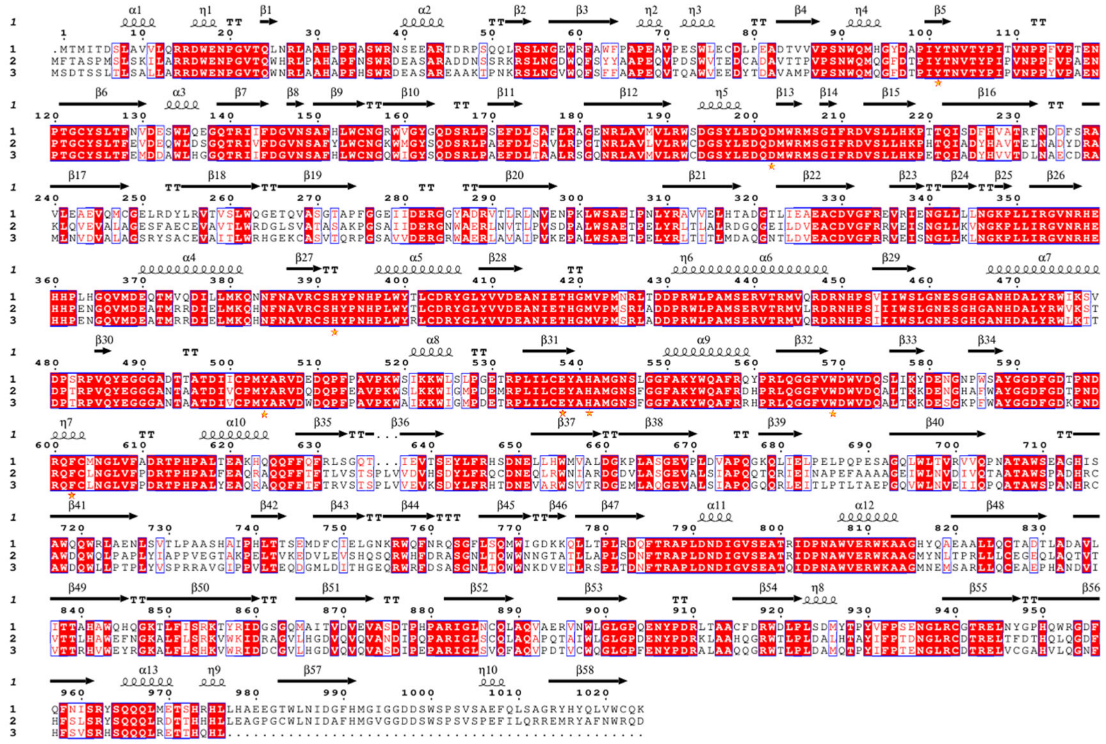

Figure S1. Multiple sequence alignment of Pstr *β*-galactosidase with selected representative *β*-galactosidases. Sequence 1 corresponds to Escherichia coli *β*-galactosidase (NCBI accession: WP_000177906.1), Sequence 2 represents Pstr *β*-galactosidase (NCBI accession: MBC3295486.1), and Sequence 3 is derived from Enterobacter cloacae *β*-galactosidase (NCBI accession: WFG05994.1). Red asterisks indicate conserved catalytic residues.

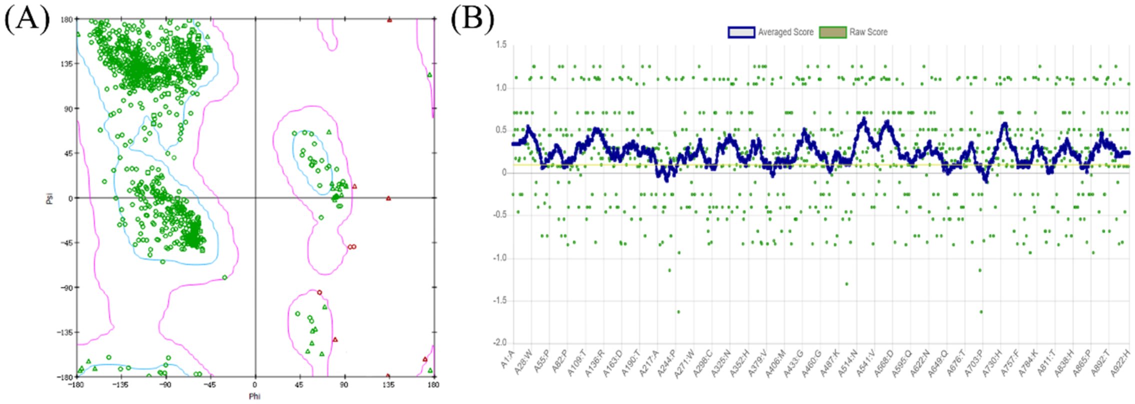

Figure S2 The accuracy evaluation of Pstr *β*-galactosidase modelling. (A) Ramachandran plot analysis of Pstr *β*-galactosidase modelling (B) Verify3D analysis of Pstr *β*-galactosidase modelling.

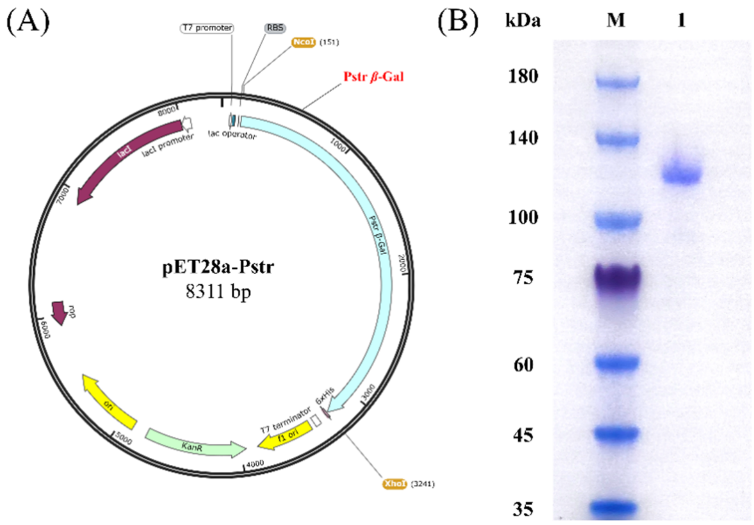

Figure S3 The gene map of the recombinant plasmid pET28a-Pstr. (B) SDS-PAGE analysis of Pstr *β*-galactosidase. Lane M, protein markers; lane 1, the purified recombinant Pstr *β*-galactosidase.

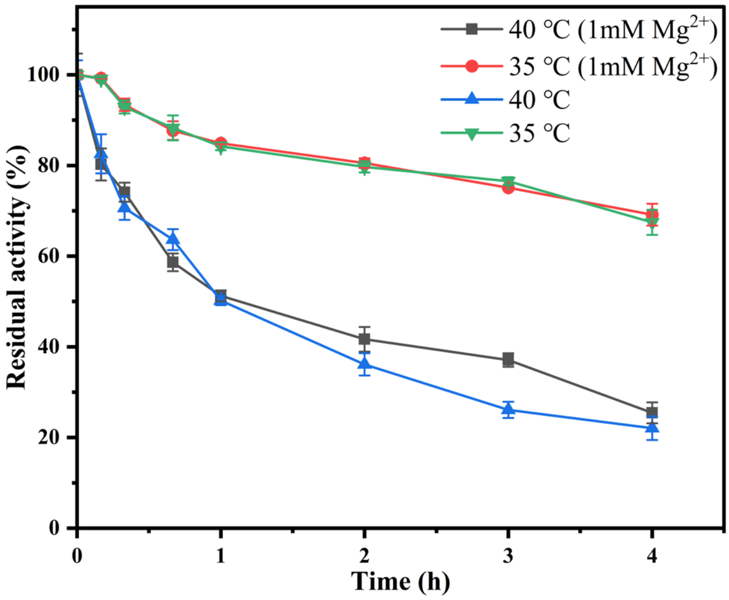

Figure S4 The influence of the metal ion (Mg^{2+}) on the thermostability of Pstr *β*-galactosidase.
